# Identification and characterization of a novel *adenomatous polyposis coli* mutation in adult pancreatoblastoma

**DOI:** 10.18632/oncotarget.24017

**Published:** 2018-01-06

**Authors:** Shigeo Yamaguchi, Tomoaki Fujii, Yuki Izumi, Yuki Fukumura, Min Han, Hideki Yamaguchi, Tomomi Akita, Chikamasa Yamashita, Shunsuke Kato, Takao Sekiya

**Affiliations:** ^1^ Department of Clinical Oncology, Juntendo University Graduate School of Medicine, Hongo, Bunkyo-ku, Tokyo, Japan; ^2^ Department of Cancer Genome Research, Sasaki Institute, Sasaki Foundation, Kandasurugadai, Chiyoda-ku, Tokyo, Japan; ^3^ Department of Pharmaceutics and Drug Delivery, Faculty of Pharmaceutical Sciences, Tokyo University of Science, Yamazaki, Noda, Chiba, Japan; ^4^ Department of Human Pathology, Faculty of Medicine, Juntendo University, Hongo, Bunkyo-ku, Tokyo, Japan; ^5^ Department of Cancer Cell Research, Sasaki Institute, Sasaki Foundation, Kandasurugadai, Chiyoda-ku, Tokyo, Japan; ^6^ Fusion of Regenerative Medicine with DDS, Research Institute for Science and Technology, Tokyo University of Science, Yamazaki, Noda, Chiba, Japan; ^7^ Division of Translational Genomics for Intractable Diseases, Intractable Diseases Research Center, Juntendo University, Hongo, Bunkyo-ku, Tokyo, Japan

**Keywords:** next generation sequencing, variant of uncertain significance, pancreatoblastoma, adenomatous polyposis coli, Wnt/β-catenin signaling

## Abstract

During next generation sequencing (NGS) analysis, many missense mutations were found in a well-known oncogene, many of which were variant of uncertain significance mutations. We recently treated an adult patient with pancreatoblastoma by chemotherapy. Using an NGS cancer panel, we found a previously unreported missense mutation in the 1835 codon of the *adenomatous polyposis coli* (*APC*) gene. We also found a heterogeneous mutation in the 1835 codon of the *APC* gene in the patient's germline by Sanger sequencing. Although this patient did not have a history of familial adenomatous polyposis, functional analysis suggested the R1835G mutant *APC* showed attenuated repression of Wnt/β-catenin signaling activity. This is the first report showing a novel *APC* missense mutation involved in the onset of adult pancreatoblastoma.

## INTRODUCTION

Pancreatoblastoma is a rare malignant epithelial neoplasm of the pancreas with multiple lines of differentiation, including acinar differentiation and squamoid nests. Less frequently, this tumor may exhibit endocrine and ductal differentiation and may contain a distinct mesenchymal component [1]. Pancreatoblastoma usually occurs in childhood and is the most prevalent malignant pancreatic tumor in the first decade of life [2]. Exceptionally, it can also be encountered in adults, although such cases are so rare that fewer than fifty cases have been reported to date [3]. No effective therapy has been established, especially in cases with unresectable or metastatic lesions. There have been only a few reports on the molecular features of pancreatoblastoma.

Recently, we encountered an adult patient with pancreatoblastoma. Using a next generation sequencer (NGS), loss of heterozygosity (LOH) of the *adenomatous polyposis coli (APC)* gene was detected in the tumor. In the remaining *APC* allele, we observed a novel missense mutation, derived from the germline *APC* gene, which was not reported in the Universal Mutation Database (http://www.umd.be/) or Tohoku Medical Megabank Organization, a Japanese-specific genome cohort (http://www.megabank.tohoku.ac.jp/). In this report, we characterized the function of the novel *APC* mutant.

## CASE PRESENTATION

**Figure 1:**
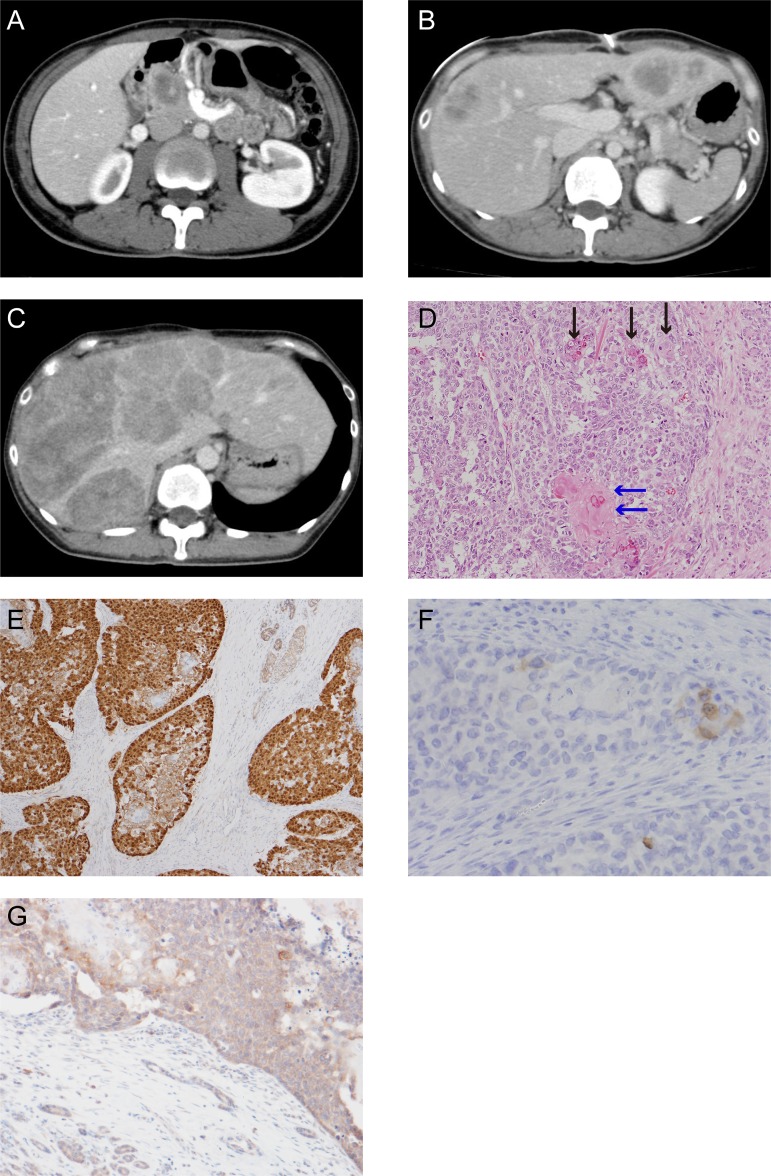
CT scan images and histological appearance of this case (**A**) CT scan image at first visit. (**B**) CT scan image at postsurgical recurrence of liver metastasis. (**C**) The last CT scan image before the patient died. (**D**) Hematoxylin and eosin staining (magnification, ×200). The black arrow shows the squamoid nest and the blue arrow shows the squamoid nest with keratinization. (**E**) Immunohistochemical staining for β-catenin (magnification, ×100). (**F**) Scattered tumor cell shows positive for Bcl-10 (magnification, ×200). (**G**) Immunohistochemical staining for APC (magnification, ×50).

A 37-year-old woman visited our hospital complaining of abdominal pain. She had no past or familial history of neoplasms. A computed tomography (CT) scan showed a 5 cm abnormal mass in the pancreas head. She was suspected for pancreatoblastoma by CT-guided biopsy (Figure 1A) and was sent for a pancreaticoduodenectomy.

The postoperative pathological findings showed squamoid nests, some with keratinization (Figure 1D). Immunohistological analysis indicated an abnormal nuclear accumulation of β-catenin proteins and the expression of Bcl-10, a pancreatic acinar cell marker, in the tumor tissues (Figures 1E and 1F). A pathological diagnosis of pancreatoblastoma depends on its acinar differentiation and on the existence of squamoid nests; therefore, this tumor was finally diagnosed as pancreatoblastoma according to these findings.

A CT scan taken three months postoperatively showed metastatic recurrence in the liver (Figure 1B). She received one course of adriamycin and gemcitabine chemotherapy, with no response. We treated her with cisplatin and S-1 as a second line chemotherapy. Although the tumor temporarily exhibited no change in size, after six cycles of cisplatin and S-1 chemotherapy her liver metastatic pancreatoblastoma increased in size (Figure 1C). We were unable to achieve any response via salvage treatment, and she ultimately died of pancreatoblastoma progression 13 months after diagnosis.

## RESULTS

### Analysis of oncodriver mutations in a patient with pancreatoblastoma

**Figure 2:**
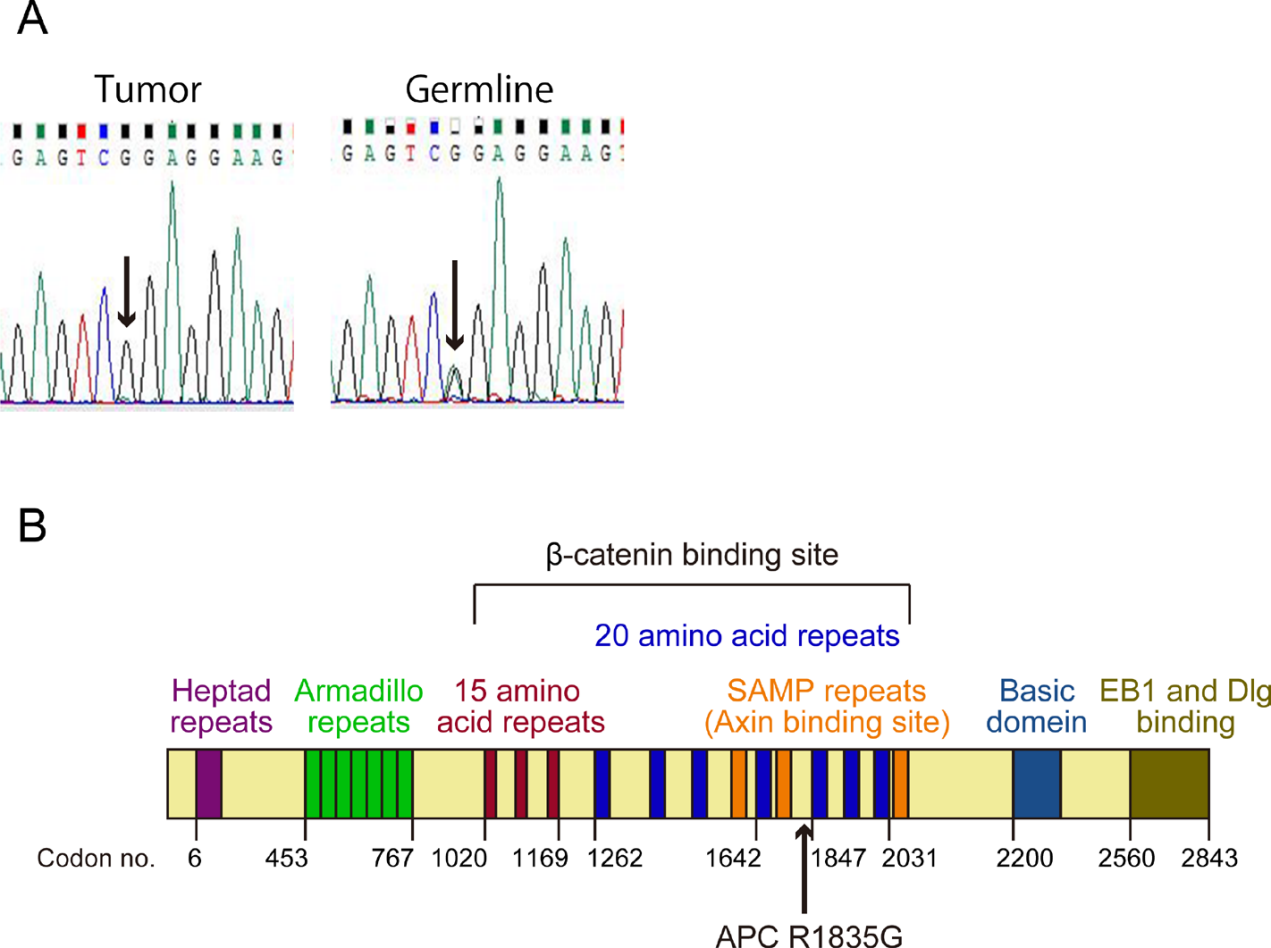
Status and position of the *APC* mutation (**A**) The results of direct sequencing of the PCR product from a part of *APC* exon 15 in the tumor and the germline. The arrows show a point mutation of c.5503A > G (p.R1835G). (**B**) Structure of APC protein. The arrow shows position of the mutation (p.R1835G).

We conducted genetic analysis to investigate genetic alterations in the tumor using a cancer panel (OncoDEEP^®^, Supplementary Table 1). We identified a previously unreported missense APC mutation of c.5503A > G (p.R1835G). We also confirmed the mutation of the *APC* gene by Sanger sequencing (Figure 2A) and the expression of APC protein by immunohistochemical staining (Figure 1G) in tumor tissue samples from the patient. Allele frequency of this mutated allele was 99% in the cancer panel analysis. In addition, we did not find any other alterations of the genes involving the Wnt signal pathway, including *CTNNB1*. After obtaining written informed consent from the patient, we analyzed her germ line *APC* gene from her saliva and found a heterogeneous mutation in the 1835 codon of the *APC* gene (Figure 2A). These data suggest that a LOH of the *APC* gene occurred in the tumor, and the products of the *APC* missense mutation took part in tumorigenesis.

### Evaluation of the suppression ability of the Wnt/β-catenin signaling pathway by R1835G mutant APC

**Figure 3:**
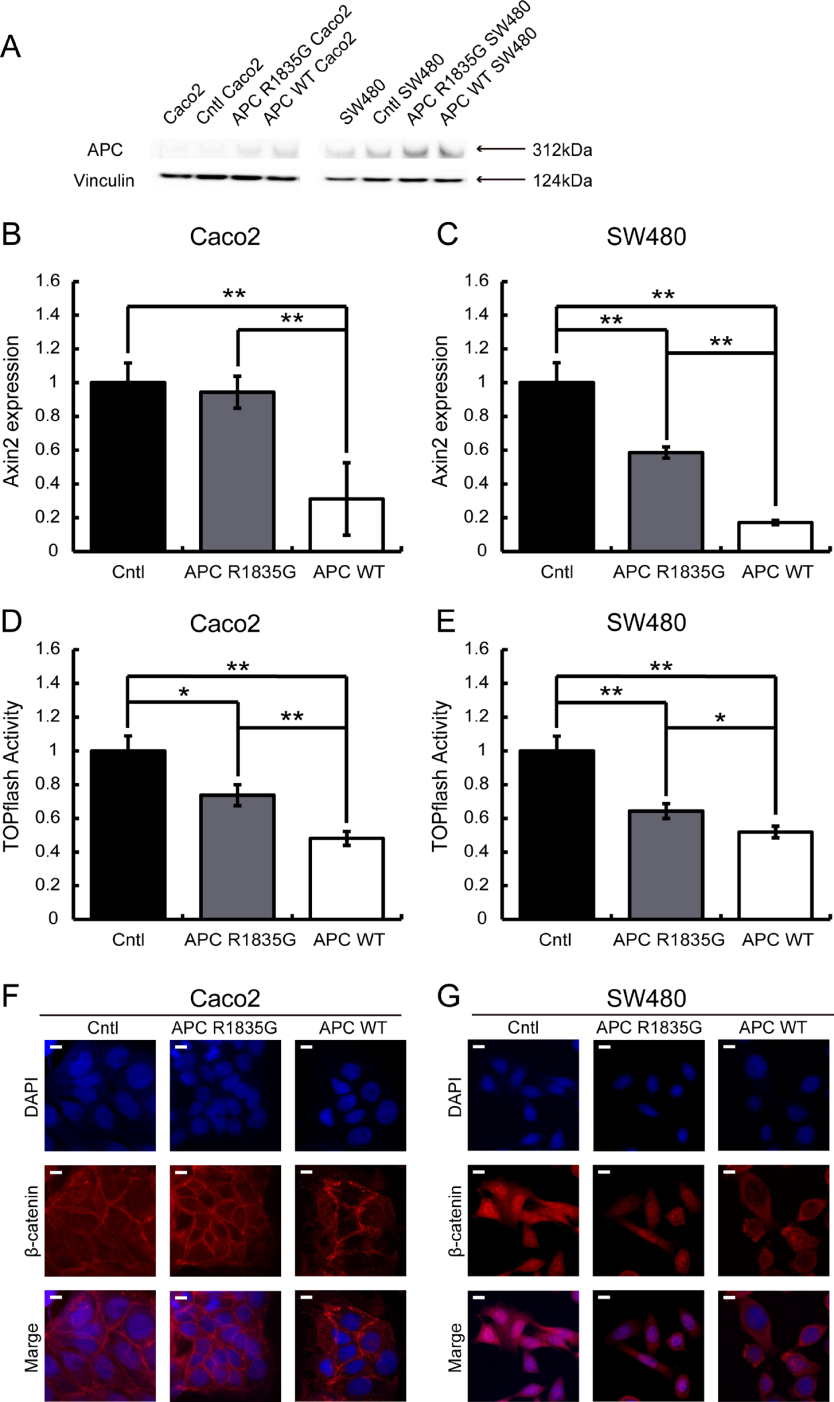
Evaluation of the suppression ability of the Wnt/β-catenin signaling pathway using a stable transformant Western blotting analysis of APC in Caco2 and SW480 cells with three genotypes (*APC*-WT, *APC*-R1835G and Cntl) were shown (**A**). Relative expression level of *AXIN2* mRNA with three genotypes in Caco2 (**B**) and SW480 (**C**) cells and activity of the TOPFlash assay with three genotypes in Caco2 (**D**) and SW480 (**E**) cells were indicated. Vertical axis of the result (B) and (C) shows relative *AXIN2* expression and (D) and (E) show relative TOPflash activity. Data are shown as the mean ± SD, ^*^p < 0.05 ^**^p < 0.01 (Student's *t*-test), and all assays were performed in triplicate. Immunofluorescence of β-catenin with three genotypes in Caco2 (**F**) and SW480 (**G**) cells were shown. Scale bar shows 10 μm.

Immunohistochemical analysis showed an abnormal nuclear accumulation of β-catenin protein (Figure 1E), indicating activation of the Wnt/β-catenin signaling pathway in the pancreatoblastoma. Therefore, to examine whether R1835G mutant *APC* affects Wnt/β-catenin signaling, we established cell models expressing APC R1835G mutant protein stably (Figure 3A). We performed quantitative RT-PCR (q-RT-PCR) to measure the endogenous Wnt/β-catenin transcriptional target, *AXIN2,* with three genotypes in Caco2 (*APC*-WT-Caco2, *APC*-R1835G-Caco2, Cntl-Caco2) and SW480 (*APC*-WT-SW480, *APC*-R1835G-SW480, Cntl-SW480). The q-RT-PCR analysis indicated that expression of *AXIN2* in *APC* R1835G was reduced compared to Control (Cntl), but its expression was not downregulated as much as in *APC*-WT (Figure 3B and 3C). To examine how *APC* R1835G affects the Wnt/β-catenin signaling pathway via the TCF/LEF binding site, we carried out a TOPFlash assay with three genotypes in Caco2 and SW480 (Figure 3D and 3E). Similar results were also obtained from the TOPFlash assay. These results showed that a R1835G mutation in the *APC* gene partially inhibits Wnt/β-catenin signaling activity due to the *APC* mutant directly affecting the β-catenin/TCF transcriptional complex. In addition, we performed an immunofluorescence analysis for β-catenin, comparing the nuclear accumulation of β-catenin among the three genotypes in Caco2 and SW480. As can be seen in Figure 3F and 3G, there was greater accumulation of β-catenin in the nucleus with APC R1835G expressing cell lines than with APC-WT expressing cell lines. It is suggested that the product of APC R1835G has weak ability to bind β-catenin compared with WT.

### Evaluation of the suppression ability of the Wnt/β-catenin signal by R1835G mutant APC with external Wnt stimuli

**Figure 4:**
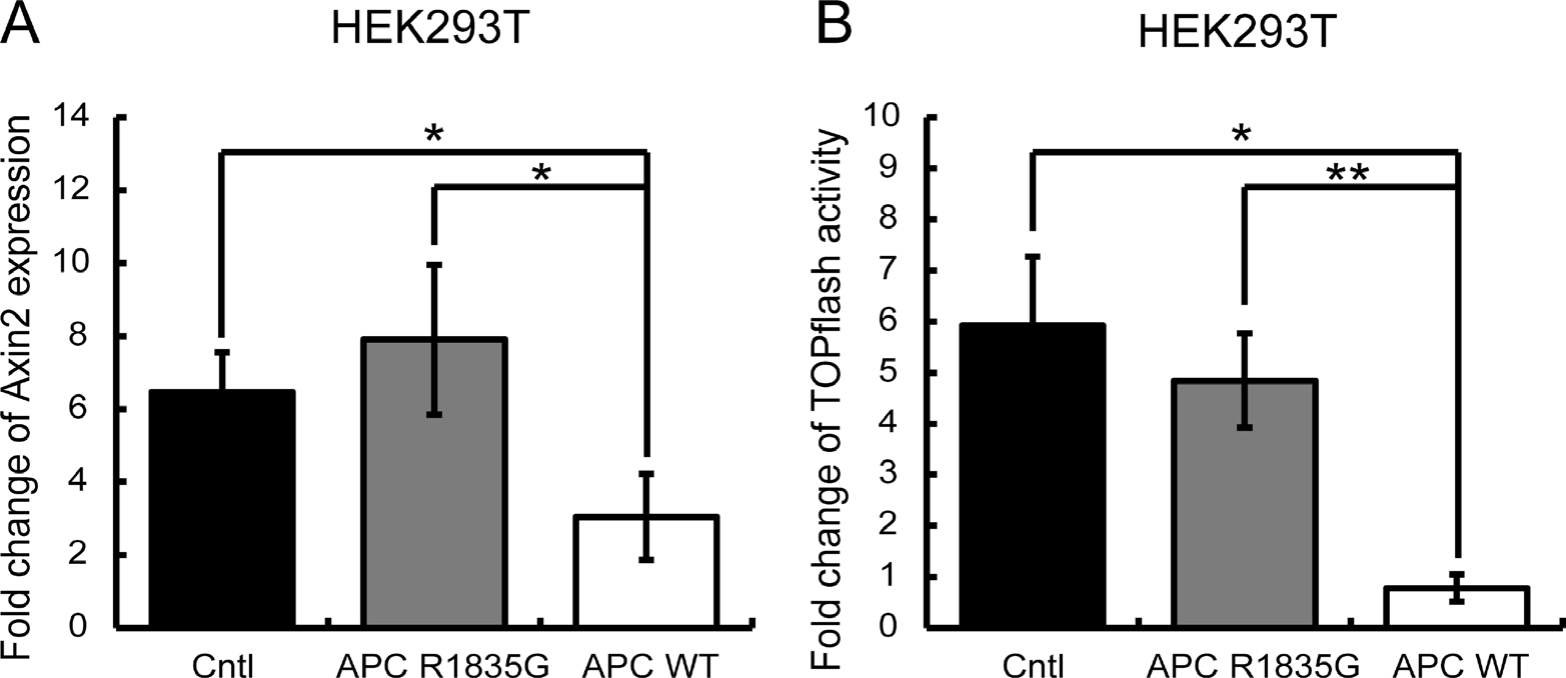
Evaluation of the suppressive ability of the Wnt/β-catenin signaling pathway under the condition of external Wnt signaling stimuli (**A**) Relative expression levels of *AXIN2* mRNA with three genotypes in HEK293T (*APC*-WT-HEK293T, *APC*-R1835G-HEK293T, and Cntl-HEK293T) cells are shown. (**B**) Activities of the TOPFlash assay with three genotypes in HEK293T were indicated. Vertical axis of result (A) shows fold change of *AXIN2* expression and the result (B) shows fold change of TOPflash activity before and after LiCl stimulation. Data are shown as the mean ± SD, ^*^p < 0.05 ^**^p < 0.01 (Student's *t*-test), and all assays were performed in triplicate.

Next, we evaluated whether the R1835G mutant *APC* had the ability to suppress activated Wnt/β-catenin signaling in HEK293T cells, which originally have a wild-type *APC* allele. The activation of the Wnt/β-catenin pathway was mimicked by the inhibition of GSK-3β using the pharmacological inhibitor LiCl (lithium chloride). LiCl was added 6 h before the assay. As shown in Figure 4, activation of Wnt/β-catenin signaling was suppressed under the stably overexpressed *APC* WT. However, the stably overexpressed *APC* R1835G could not completely suppress activation of Wnt/β-catenin signaling. These results show that the R1835G mutant *APC* can partially, but not fully, inhibit Wnt/β-catenin signaling activity.

### Dose-dependent assessment of suppression ability of the Wnt/β-catenin signaling pathway by R1835G mutant APC

**Figure 5:**
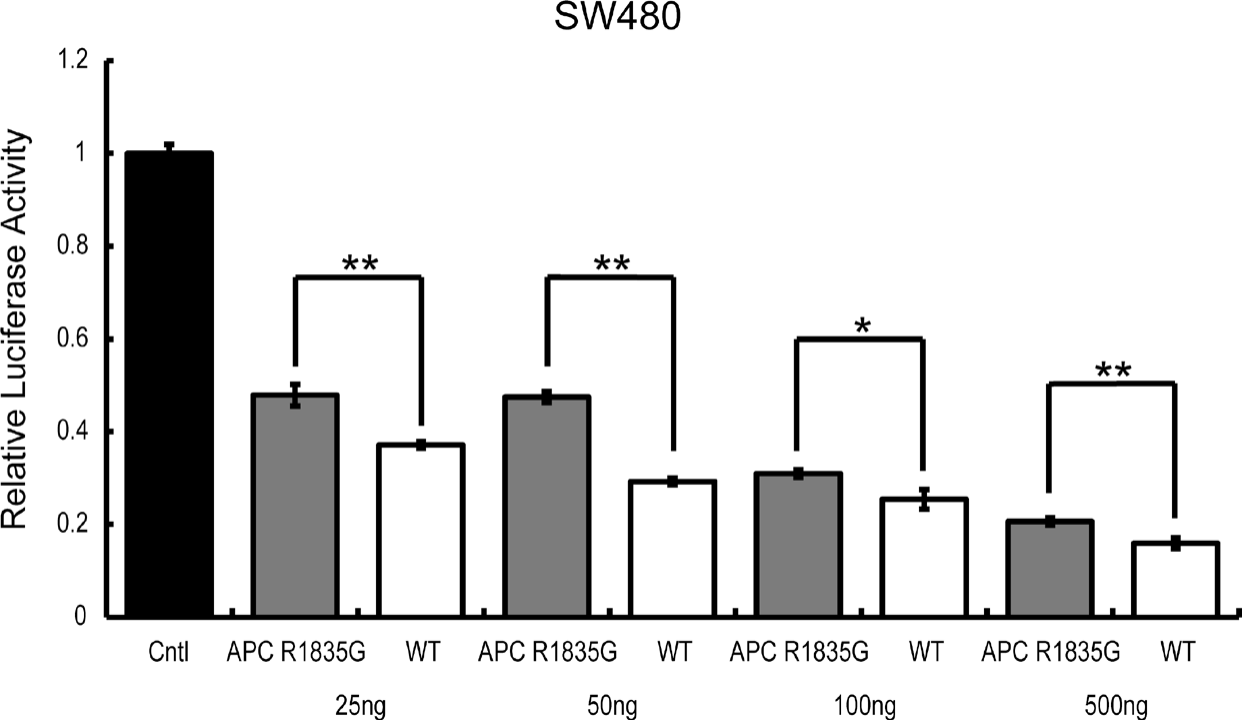
TOPFlash reporter activity by *APC*-R1835G expression levels in SW480 TOPflash activity was assessed after transfection of pCMV-Neo-Bam *APC*-R1835G and pCMV-Neo-Bam *APC* in SW480 cells. Each plasmid is transfected at 25 ng, 50 ng, 100 ng, and 500 ng. Data are shown as the mean ± SD, ^*^p < 0.05 ^**^p < 0.01 (Student's *t*-test), and all assays were performed in triplicate.

To determine if the suppression ability of Wnt/β-catenin signaling by *APC* R1835G was dose-dependent, we conducted a TOPFlash assay using ectopically expressed *APC* R1835G in SW480. Expression of increasing amounts of *APC* R1835G reduced TOPFlash reporter activity in a dose-dependent manner. We observed enhanced TOPFlash reporter activity of *APC* R1835G, compared to *APC* WT (Figure 5). These results show that R1835G mutant *APC* attenuate repression of the Wnt/β-catenin signaling pathway activity.

## DISCUSSION

This is a case report of pancreatoblastoma in an adult patient. Pancreatoblastoma is a very uncommon neoplasm in adults, and its molecular pathogenesis is largely unknown. We found a novel missense mutation in the *APC* gene. Although the tumor cells had only novel missense mutation alleles of the *APC* gene, germ cell lines had wild-type and mutant alleles of *APC*. Moreover, neoplasms showed abnormal nuclear accumulation of β-catenin protein by immunohistological analysis. Therefore, this case of pancreatoblastoma was likely caused by activation of the Wnt/β-catenin signal by LOH of the *APC* gene.

According to the cBioPortal for Cancer Genomes (http://www.cbioportal.org/), somatic mutations in the *APC* and *CTNNB1* loci have rarely been identified in pancreatic cancer, but a few *CTNNB1* gene mutations have been reported in cases of pancreatoblastoma [4–7]. Because of the rarity of pancreatoblastoma, the population of *APC* gene mutations is not known. Only one case has been reported of a germline truncating mutation of the *APC* gene, a 5-bp deletion at the β-catenin binding site in an individual with familial adenomatous polyposis (FAP) [6]; LOH of the *APC* gene in pancreatoblastoma was also observed in this case (Table 1). Both pancreatoblastoma and acinar cell carcinomas consistently exhibit acinar differentiation [1], as well as alterations in the genes for Wnt/β-catenin signaling, including inactivating mutations in *APC* and activating mutations in *CTNNB1*; however, they lack alterations in the genes commonly mutated in pancreatic duct adenocarcinoma, including *KRAS*, *TP53*, *P16/CDKN2A*, and *SMAD4* [8]. We have therefore discussed the activation of Wnt/β-catenin signaling in the pancreas cause not ductal cancer but acinar cell carcinoma, including pancreatoblastoma. This is consistent with previous reports of alterations in the genes for Wnt/β-catenin signaling, such as *APC* and *CTNNB1*, in pancreatoblastoma (Table 1).

**Table 1:** Mutation profiles of *CTNNB1* and *APC* in pancreatoblastoma reported in the past

Case	Age/Sex	CTNNB1 alternations	APC alternations	Author
1	5/Male	S37P	Not evaluated	Tanaka *et al.*(4)
2	3/Male	S33P	Not evaluated	
3	5/Male	N32H	Not evaluated	Abraham *et al.*(6)
4	3/Male	Wild type	Not evaluated	
5	4/Male	S33F	Not evaluated	
6	45/Female	S37F	Not evaluated	
7	51/Female	Wild type	Germline	
8	6/Male	Wild type	Wild type	
9	13/Female	Wild type	Wilde type	
10	5/Female	G34R	Not evaluated	
11	3.5/Female	N32H	Not evaluated	
12	3 day/Male	T44A	Not evaluated	Ismale *et al.*(7)

*APC* regulates turnover of cytosolic β-catenin and is the key effector of the canonical Wnt signal pathway. The nucleotide sequence of the *APC* gene is conserved across species, and *APC* gene mutations exhibit closely related oncogenesis. The position of the mutation on the *APC* gene is also important because this gene carries a variety of functional domains, including a heptad repeat, an Arm repeat, a β-catenin binding site (three 15-amino acid repeats and seven 20-amino acid repeats), an *Axin* binding site (SAMP repeats), a basic region, and an EB1 binding site [9]. The mutation we report here is located between the fourth and fifth 20-amino acid repeat in the β-catenin binding site (Figure 2B). The mutation, which caused an amino acid to change from hydrophobic arginine to hydrophilic glycine, was located very close to the β-catenin binding domain. Given this, we hypothesized that this mutation may reduce the ability to bind to β-catenin. This hypothesis was supported by the result of the immunofluorescence analysis of β-catenin (Figures 3F and 3G).

*APC* germline mutations are often seen in patients with FAP, with germline truncating mutations frequently observed at the β-catenin binding site, such as in Case 7 in Table 1. In contrast, our patient had no family history of FAP or past history of colon polyposis. This indicated that the mutation of the *APC* gene in this case caused pancreatoblastoma, not adenomatous polyposis. Another report described gastric polyposis caused by a missense mutation in the 5′ terminal side of *APC* [10]; and Ikenoue *et al.* reported that a truncated mutation at the 3′ terminal side of the *APC* gene caused attenuated polyposis [11]. In addition, there has been a report of *APC* missense mutations in cases of hepatoblastoma; however, these cases all featured childhood disease onset, and we were unable to determine whether the patients developed adenomatous polyposis [12].

As described above, in this case the mutation seemed to result in partial loss of biding ability to β-catenin. Differences in the ability to suppress Wnt/β-catenin signaling may account for the phenotypic differences that determine whether adenomatous polyposis occurs. In future studies, we need to investigate the relationship between phenotypes and mutations under *in vivo* conditions.

Comprehensive analysis by NGS gave us a lot of information. On the other hand, we found many variant of uncertain significance (VUS) mutations. There is no established method to confirm if mutations found by clinical sequencing have pathological significance or not. This case report suggests a desperate need for an established evaluation method of VUS mutations in the NGS era.

## MATERIALS AND METHODS

### Mutation analysis by cancer panel and Sanger sequence

The gene analysis of tumor cells was performed using the OncoDEEP Clinical^®^ (OncoDNA, Belgium), a cancer panel for 409 kinds of genes by NGS. Supplementary Table 1 shows the 409 gene varieties that are detected by OncoDEEP Clinical^®^. We submitted five tumor tissue sections with 5 μm thickness and one hematoxylin and eosin staining section to the OncoDNA Company. Almost all exons of the *APC* and *CTNNB1* genes were covered: for *APC*, exons 1–10, 12, 15, and 16 were 100% covered, exons 13 and 14 were more than 90% covered, and exon 11 was more than 30% covered; for *CTNNB1*, exons 1–3, 6–9, and 11–15 were 100% covered, and exons 4, 5, and 10 were more than 90% covered.

To confirm the mutation detected by OncoDEEP Clinical^®^, we performed Sanger sequencing of the *APC* gene using a primer, 5′-TCGTCTTCTGCACCCAACAA–3′. For Sanger sequencing of the *APC* gene from the germline, genomic DNA was extracted from saliva using the Oragene DNA^®^ (DNA genotek, ON, Canada).

### Immunohistochemical staining

Five micron sections of paired neoplastic and normal formalin-fixed, paraffin-embedded tissues were used for immunohistochemical staining with anti-β-catenin (clone β-catenin-1), anti-Bcl10 (clone sc-5273) and anti-APC (clone GTX116009) listed in Supplementary Table 2, according to the manufacturers’ instructions. For β-catenin, immunohistochemical labeling was evaluated for the presence of nuclear, cytoplasmic, and membranous β-catenin accumulation in both the neoplasms and the normal surrounding tissues.

### Plasmid construction

We used pCMV-Neo-Bam *APC*, which was a gift from Bert Vogelstein (Addgene plasmid # 16507)] as a template of *APC* [13]. DNA fragments of *APC* were amplified by PCR using a set of primers, 5′-CGAGGTTAACGAATTATGGCTGCAGCTTCATATG-3′ and 5′-CTACCCGGTAGAATTTTAAACAGATGTCACAAGGTA-3′. Amplified *APC* fragments were subcloned into EcoRI-digested pMSCV PIG, which was a gift from David Bartel (Addgene plasmid # 21654) [14], using an In-Fusion^®^ HD cloning kit (Takara). The constructed plasmid containing *APC* WT was named pMSCV-*APC*-WT.

Plasmids containing *APC* R1835G (c.*5503a > g*) were generated from pMSCV-*APC*-WT using site-directed mutagenesis and named pMSCV-*APC*-R1835G. The primers used for site-directed mutagenesis were 5′-GAAGATAGAGTCGGAGGAAGTTTTG-3′ and 5′-CAAAACTTCCTCCGACTCTATCTTC-3′.

### Cell lines and culture

Colorectal cancer cell lines Caco-2 and SW480 (*APC*-null) and embryonic kidney cell lines HEK293T (*APC* wild type) were cultured according to recommendations of the American Type Culture Collection (ATCC). PLAT-A cells [15] were maintained at 37°C in a 5% CO_2_ incubator in DMEM, 10% fetal calf serum (FCS), 1 μg/mL puromycin, 10 μg/mL blasticidin, and penicillin.

### Retroviral transduction and establishment of a stable transformant

The day before transfection, PLAT-A cells, at 2 × 10^6^ per dish, were seeded in a 60 mm culture dish and incubated overnight. Each of pMSCV-*APC*-WT, pMSCV-*APC*-R1835G and pMSCV PIG was transfected into PLAT-A cells at 3 μg using GeneJuice Transfection Reagent (Novagen). Retrovirus particles from PLAT-A cells were collected 48 h after transfection and added to Caco-2, SW480, and HEK293T cells with 8 μg/ml Polybren (Sigma). We generated *APC*-WT-Caco2, *APC*-R1835G-Caco2, Cntl-Caco2, *APC*-WT-SW480, *APC*-R1835G-SW480, Cntl-SW480, *APC*-WT-HEK293T, *APC*-R1835G-HEK293T, and Cntl-HEK293T cells. After infection, Caco-2, SW480 and HEK293T cells were incubated with the 8 ng/μl, 4 ng/μl, and 1.5 ng/μl puromycin selection agents, respectively, to obtain stable cell lines.

### Western blotting

Cellular lysates were prepared and the protein concentrations were determined using a Pierce^TM^ BCA Protein Assay Kit (Thermo Fisher Scientific). Samples with 13 μg and 5 μg of total protein were used for western blots of Caco-2 and SW480, respectively. The samples were separated via electrophoresis on 5%–12% SDS-polyacrylamide gel and then transferred onto a nylon membrane for the western blot analysis. The membrane was probed with the polyclonal rabbit antibodies listed in Supplementary Table 2, anti-APC (clone ab15270), and anti-Vinculin (clone ab73412) as the loading control. Anti-rabbit IgG.

HRP-linked Antibody (Cell Signaling Technology) was used as the secondary antibody. The protein bands were detected using Pierce^®^ ECL Plus Western Blotting Substrate (Thermo Fisher Scientific).

### Transient transfection

We generated pCMV-Neo-Bam *APC*-R1835G from pCMV-Neo-Bam *APC* by site-directed mutagenesis using a set of primers, 5′- GAAGATAGAGTCGGAGGAAGTTTTG-3′ and 5′- CAAAACTTCCTCCGACTCTATCTTC-3′. The day before transfection, SW480 cells, at 2 × 10^5^ cells per dish, were seeded in a 60 mm culture dish and incubated overnight. Each of pCMV-Neo-Bam *APC*-R1835G, pCMV-Neo-Bam *APC*, and pCMV were transfected into SW480 cells at 25 ng, 50 ng, 100 ng, and 500 ng using GeneJuice Transfection Reagent (Novagen).

### Luciferase assay

In an assay of stable cell lines, the day before transfection for reporter gene assays, *APC*-WT-Caco2, *APC*-R1835G-Caco2, Cntl-Caco2, *APC*-WT-SW480, *APC*-R1835G-SW480, Cntl-SW480, *APC*-WT-HEK293T, *APC*-R1835G-HEK293T, and Cntl-HEK293T cells, at 2 × 10^5^ per dish, were seeded in six-well plates. Each well was transfected with a total of 78 ng of plasmids, including 65 ng of M50 Super 8× TOPFlash, which was a gift from Randall Moon (Addgene plasmid # 12456) [16], or M51 Super 8× FOPFlash, also a gift from Randall Moon (Addgene plasmid # 12457) [16], and 13 ng of pRL-SV40P, which was a gift from Ron Prywes (Addgene plasmid # 27163) [17], with GeneJuice Transfection Reagent (Novagen). Luciferase activity was monitored by SpectraMax^®^i3 (MOLECULAR DEVICES) using the dual luciferase assay system (Promega). We controlled the experimental LEF-luciferase reporter activity for transfection efficiency and potential treatment toxicity using the constitutively expressed pRL-SV40P luciferase. The specificity of *APC*-mediated effects on LEF reporters was confirmed using the M51 Super 8× FOPFlash, which harbors mutated LEF binding sites and an unrelated AP-1 reporter. Concerning to *APC*-WT-HEK293T, *APC*-R1835G-HEK293T, and Cntl-HEK293T cells, LiCl was added as a stimulant 6 h before the luciferase assay. In assay of transiently transfected SW480 cells, each of M50 Super 8× TOPFlash, M51 Super 8× FOPFlash, and pRL-SV40P were transfected in conjunction with transfection of pCMV-Neo-Bam *APC*-R1835G, pCMV-Neo-Bam *APC*, and pCMV.

### Quantitative RT-PCR

The day before RNA extraction, *APC*-WT-Caco2, *APC*-R1835G-Caco2, Cntl-Caco2, *APC*-WT-SW480, *APC*-R1835G-SW480, Cntl-SW480, *APC*-WT-HEK293T, *APC*-R1835G-HEK293T, and Cntl-HEK293T cells, at 2 × 10^5^ cells per dish, were seeded in six-well plates. Total RNAs were extracted from each well of these cells with NucleoSpin RNA (MACHEREY-NAGEL). Quantification of *AXIN2* gene transcripts was performed by quantitative RT-PCR (q-RT-PCR) using the GoTaq 1-Step RT-qPCR System (Promega). *HPRT1* was used as an internal control. The primer pairs used for the human *AXIN2* gene were 5′-CTGGCTTTGGTGAACTGTTG-3′ and 5′-AGTTGCTCACAGCCAAGACA-3′. The primer pair used for the human *HPRT1* gene was 5′-GC ACCACCAACTGCTTA-3′ and 5′-AGTAGAGGCAGG GATGAT-3′. Concerning *APC*-WT-HEK293T, *APC*-R183 5G-HEK293T, and Cntl-HEK293T cells, LiCl was added as stimulant 6 h before RNA extraction.

### Immunofluorescence

Caco2 and SW480 cells grown and transfected onto 12-well chambers were fixed for 5 min in 10% methanol at −20°C. The cells were then washed three times with phosphate-buffered saline (PBS) and incubated with 1% bovine serum albumin (BSA) in PBS for 30 min at room temperature. Anti-β-catenin (clone 14/β-catenin) antibody in 1% BSA was then added to the samples, which were incubated overnight at 4°C. After washing three times with PBS, the cells were treated with Goat Anti-Mouse IgG H&L (Alexa Fluor^®^ 594; Abcam) as the secondary antibody, diluted at 1:200 in 1% BSA for 1 h at room temperature. After washing three more times with PBS, the nuclei were counterstained with ProLong^TM^ Gold antifade reagent with DAPI (Invitrogen). The samples were observed using a BZ-X710 (Keyence).

## SUPPLEMENTARY MATERIALS TABLES





## Abbreviations

NGS: next generation sequencer
LOH: loss of heterozygosity
*APC*: *adenomatous polyposis coli*
CT: computed tomography
q-RT-PCR: quantitative RT-PCR
LiCl: lithium chloride
FAP: familial adenomatous polyposis
VUS: variant of uncertain significance
ATCC: American type culture collection
FCS: fetal calf serum
PBS: phosphate-buffered saline
BSA: bovine serum albumin
