# Supplementary material for: Identification and characterization of a novel adenomatous polyposis coli mutation in adult pancreatoblastoma
Source: Oncotarget. 2018 Jan 6;9(12):10818–27. doi: 10.18632/oncotarget.24017 (PMC5828192; doi:10.18632/oncotarget.24017)
Supplement: Supplementary file 1 [file oncotarget-09-10818-s001.pdf]

Identification and characterization of a novel *adenomatous polyposis coli* mutation in adult pancreatoblastoma

SUPPLEMENTARY MATERIALS

Supplementary Table 1: 409 genes list detected in the OncoDEEP® Clinical. See Supplemenatary_Table_1

Supplementary Table 2: Antibody used for immunohistochemical staining and immunofluorescence

Antibody	Clone	Dilution	Pretreatment	Source
β-catenin	β-catenin-1	1:200	CB	DAKO/Agilent, CA, USA
β-catenin	14/β-catenin	1:100		BD Biosciences CA, USA
Bcl-10	sc-5273	1:400	CB	Santa Cruz, CA, USA
APC	GTX116009	1:200	CB	GeneTex CA, USA
APC	ab15270	1:500		abcam Cambs. UK
Vinculin	Ab73412	1:500		abcam Cambs. UK

CB; Citrate buffer (PH6.0).
