# Supplementary material for: Identification and characterization of a novel adenomatous polyposis coli mutation in adult pancreatoblastoma
Source: Oncotarget. 2018 Jan 6;9(12):10818–27. doi: 10.18632/oncotarget.24017 (PMC5828192; doi:10.18632/oncotarget.24017)
Supplement: Supplementary file 2 [file oncotarget-09-10818-s002.docx]

Table S1. 409 genes list detected in the OncoDEEP® CLINICAL

| ABL1 | ABL2 | ACVR2A | ADAMTS20 | AFF1 | AFF3 | AKAP9 | AKT1 |
| --- | --- | --- | --- | --- | --- | --- | --- |
| AKT2 | AKT3 | ALK | APC | AR | ARID1A | ARID2 | ARNT |
| ASXL1 | ATF1 | ATM | ATR | ATRX | AURKA | AURKB | AURKC |
| AXL | BAI3 | BAP1 | BCL10 | BCL11A | BCL11B | BCL2 | BCL2L1 |
| BCL2L2 | BCL3 | BCL6 | BCL9 | BCR | BIRC2 | BIRC3 | BIRC5 |
| BLM | BLNK | BMPR1A | BRAF | BRD3 | BRIP1 | BTK | BUB1B |
| CARD11 | CASC5 | CBL | CCND1 | CCND2 | CCNE1 | CD79A | CD79B |
| CDC73 | CDH1 | CDH11 | CDH2 | CMPK1 | CDH20 | CDH5 | CDK12 |
| CDK4 | CDK6 | CDK8 | CDKN2A | CDKN2B | CDKN2C | CEBPA | CHEK1 |
| CHEK2 | CIC | CKS1B | COL1A1 | CRBN | CREB1 | CREBBP | CRKL |
| CRTC1 | CSF1R | CSMD3 | CTNNA1 | CTNNB1 | CYLD | CYP2C19 | CYP2D6 |
| DAXX | DCC | DDB2 | DDIT3 | DDR2 | DEK | DICER1 | DNMT3A |
| DPYD | DST | EGFR | EML4 | EP300 | EP400 | EPHA3 | EPHA7 |
| EPHB1 | EPHB4 | EPHB6 | ERBB2 | ERBB3 | ERBB4 | ERCC1 | ERCC2 |
| ERCC3 | ERCC4 | ERCC5 | ERG | ESR1 | ETS1 | ETV1 | ETV4 |
| EXT1 | EXT2 | EZH2 | FAM123B | FANCA | FANCC | FANCD2 | FANCF |
| FANCG | FAS | FBXW7 | FGFR1 | FGFR2 | FGFR3 | FGFR4 | FH |
| FLCN | FLI1 | FLT1 | FLT3 | FLT4 | FN1 | FOXL2 | FOXO1 |
| FOXO3 | FOXP1 | FOXP4 | FZR1 | G6PD | DATA1 | HRAS | GATA2 |
| GATA3 | GDNF | GNA11 | GNAQ | GNAS | GPR124 | GRM8 | GUCY1A2 |
| HCAR1 | HIF1A | HLF | HNF1A | HOOK3 | HSP90AA1 | HSP90AB1 | ICK |
| IDH1 | IDH2 | IGF1R | IGF2 | IGF2R | IKBKB | IKBKE | IKZF1 |
| IL2 | IL21R | IL6ST | IL7R | ING4 | IRF4 | IRS2 | ITGA10 |
| ITGA9 | ITGB2 | ITGB3 | JAK1 | JAK2 | JAK3 | JUN | KAT6A |
| KAT6B | KDM5C | KDM6A | KDR | KEAP1 | KIT | KLF6 | KMT2A |
| KMT2C | KMT2D | KRAS | LAMP1 | LCK | LIFR | LPHN3 | LPP |
| LRP1B | LTF | LTK | MAF | MAFB | MAGEA1 | MAGI1 | MALT1 |
| MAML2 | MAP2K1 | MAP2K2 | MAP2K4 | MAP3K7 | MAPK1 | MAPK8 | MARK1 |
| MARK4 | MBD1 | MCL1 | MDM2 | MDM4 | MEN1 | MET | MITF |
| MLH1 | MLLT10 | MMP2 | MN1 | MPL | MRE11A | MSH2 | MSH6 |
| NCOA2 | MTOR | MTR | MTRR | MUC1 | MUTYH | MYB | MYC |
| MYCL1 | MYCN | MYD88 | MYH11 | MYH9 | NBN | NCOA1 | NCOA4 |
| NF1 | NF2 | NFE2L2 | NFKB1 | NFKB2 | NIN | NKX2-1 | NLRP1 |
| NOTCH1 | NOTCH2 | NOTCH4 | NPM1 | NRAS | NSD1 | NTRK1 | NTRK3 |
| NUMA1 | NUP214 | NUP98 | PAK3 | PALB2 | PARP1 | PAX3 | PAX5 |
| PAX7 | PAX8 | PBRM1 | PBX1 | PDE4DIP | PDGFB | PDGFRA | PDGFRB |
| PER1 | PGAP3 | PHOX2B | PIK3C2B | PIK3CA | PIK3CB | PIK3CD | PIK3CG |
| PIK3R1 | PIK3R2 | PIM1 | PKHD1 | PLAG1 | PLCG1 | PLEKHG5 | PML |
| PMS1 | PMS2 | POT1 | POU5F1 | PPARG | PPP2R1A | PRDM1 | PRKAR1A |
| PRKDC | PSIP1 | PTCH1 | PTEN | PTGS2 | PTPN11 | PTPRD | PTPRT |
| RAD50 | RAF1 | RALGDS | RARA | RB1 | RECQL4 | REL | RET |
| RHOH | RNASEL | RNF2 | RNF213 | ROS1 | RPS6KA2 | RRM1 | RUNX1 |
| RUNX1T1 | SAMD9 | SBDS | SDHA | SDHB | SDHC | SDHD | SEPT9 |
| SETD2 | SF3B1 | SGK1 | SH2D1A | SMAD2 | SMAD4 | SMARCA4 | SMARCB1 |
| SMO | SMUG1 | SOCS1 | SOX11 | SOX2 | SRC | SSX1 | STK11 |
| STK36 | SUFU | SYK | SYNE1 | TAF1 | TAF1L | TAL1 | TBX22 |
| TCF12 | TCF3 | TCF7L1 | TCF7L2 | TCL1A | TET1 | TET2 | TFE3 |
| TGFBR2 | TGM7 | THBS1 | TIMP3 | TLR4 | TLX1 | TNFAIP3 | TNFRSF14 |
| TNK2 | TOP1 | TP53 | TPR | TRIM24 | TRIM33 | TRIP11 | TSHR |
| UBR5 | UGT1A1 | USP9X | VHL | WAS | WHSC1 | WRN | WT1 |
| XPA | XPC | XPO1 | TRRAP | TSC1 | TSC2 | XRCC2 | ZNF384 |
| ZNF521 |  |  |  |  |  |  |  |
